# Dangerous Drugs: Bromide of Potassium

**Published:** 1887-07-23

**Authors:** 


### THE DOCTOR'S PULPIT.

### DANGEROUS DRUGS.

### Bromide of Potassium.

There is not a more harmless drug in the Pharmacopœia than bromide of potassium—nor a more dangerous. A child may dispense it with safety: a Fellow of the College of Physicians may ruin a patient's health, and even destroy his life, by means of it. The therapeutic uses and advantages of the substance are universally known and appreciated by medical men: but it is to be feared that its physiological action is not always clearly recognised or adequately considered. Bromide of potash given to a healthy person contracts the minute arteries. Its action is probably exerted on the vasomotor nerves. This contracting action on the arterioles diminishes the quantity of blood in them, so long as the effect of the dose continues. By diminishing the quantity of blood circulating in the brain, the bromide acts as a sedative: that is, it lessens the activity of the brain, and so produces a soothed and quieted condition, which is very grateful after undue mental excitement. Bromide has the peculiar and not very common effect of making the patient "feel better" very quickly. A glass of good wine, or a little brandy and water, has the same effect in a different way. The wine excites and rouses up a depressed person, enabling him to throw off his depression almost at once. The bromide, on the other hand, soothes down an excited person, and enables him to control his excitement and worry. This property of producing a decided result, of which the patient himself is quickly and unmistakably conscious, constitutes the chief danger of the use of bromide, as it does of the use of alcohol. It makes the drug dangerous in the hands of the doctor, because he knows that by means of it he can give, at any rate, temporary relief to very distressing symptoms, and he is therefore often tempted to continue its use for a much longer time than is either safe or beneficial; and for the same reason it is still more dangerous to the patient who may be the possessor of a prescription with a considerable quantity of the bromide in it. The doctor knows the possible dangers, but shuts his eyes to them for the sake of the immediate pleasant result. The patient does not know the risks, and so goes on week after week in happy unconsciousness, only discovering that the dose of his comforter requires to be constantly increased.

The following cases, occurring in the writer's experience, illustrate some of the peculiar dangers arising from the too long-continued use of the bromides.

Major —— returned from India in 1877, after a prolonged residence there in worrying and exciting circumstances. He placed himself under the care of Dr. ——, who, finding him naturally nervous and preternaturally irritable as the consequence of his worries, put him on considerable doses of bromide. For a time the results were excellent, and the major used to speak of his doctor as a "fine old fellow!" Two years elapsed; health was not restored, and the bromide had become indispensable. When it was not taken the hands trembled, the brain felt "woolly" and incapable, and the temper was unbearable. The major felt sure he should never see India any more, and realised that his pension would be miserably small for himself, his wife, and family. He consulted the writer, who, finding him a perfectly sober man, with a good personal and family history, and no evidence of organic disease, examined his prescriptions, and found that he had been taking considerable doses of bromide more or less continuously for nearly two years. He ordered the patient to leave London for a tour in the North, to take no medicine of any kind, and to give the new treatment a month's trial. It was necessary that further leave should be obtained, and a certificate was sent in to the effect that "Major —— was unable to return to his duties in consequence of the highly irritable and enfeebled condition of his nervous system, due to the too long-continued use of bromide of potassium." The certificate excited some comment, but the leave was granted. Major —— discontinued his drug, went to Scotland for a month, returned to London better, walked ten or twelve miles daily for some time, and at the end of six months was certified efficient, and went back to India in excellent health; where he now is. The discontinuance of the drug for a time gave rise to very distressing nervous symptoms, which cannot be here detailed, but these gradually ceased, and the health was perfect in from three to four months.

The next case is that of a lady, Mrs. E——. She was about forty-six years of age when she first came under observation. For years she had suffered from periodical losses of considerable quantities of blood, and much ensuing mental excitability. Instead of making up the losses by suitable preparations of steel, good diet, and abundance of fresh air, treatment had always been directed to subduing the nervous symptoms by means of sedatives. A huge pile of prescriptions, extending over several years, was examined, and almost every one contained bromide in large doses. The patient when seen was suffering from lessened sensibility, and still more diminished motor-power all over the body. The mind was feeble and vacant, and there were all the evidences of a general and complete collapse. The cutting off of the bromide, and the use of quinine, phosphorus, iron, and strychnia for a time produced a marked change; but there is little doubt that the bromide was again resorted to, contrary to instructions, and at the age of 48 or 49 the patient died, in every sense, mentally and physically, a complete wreck. There is no doubt whatever in the writer's mind that this lady's death was due to the periodical losses of blood, coupled with the long-continued use of the bromides. The drug, by still further lessening the already diminished quantity of blood in the brain and cord, so lowered the nutrition of the nervous system that gradual degeneration took place; and as the result of this, all the organs of the body, not receiving their due supply of nerve-energy, failed to perform their functions, and in the end a general and complete collapse of every organ took place, and death ensued. So convinced was the writer that such was the case, that he felt it his duty to record his conviction in the death-certificate.

The object of this paper is to warn practitioners and the public against the abuse of a drug which, though apparently harmless, and in many cases of invaluable service, may yet in careless or unskilful hands prove dangerous, and even fatal. There is no doubt that some medical men place no restrictions upon the use of bromide, to the great injury and danger of their patients. And it is quite as certain that many patients foclishly and wilfully indulge in its use against the advice of the skilled adviser, and to their own serious and permanent harm.

